# Anthranilate phosphoribosyltransferase from the hyperthermophilic archaeon *Thermococcus kodakarensis* shows maximum activity with zinc and forms a unique dimeric structure

**DOI:** 10.1002/2211-5463.12264

**Published:** 2017-07-24

**Authors:** Sumera Perveen, Naeem Rashid, Xiao‐Feng Tang, Tadayuki Imanaka, Anastassios C. Papageorgiou

**Affiliations:** ^1^ School of Biological Sciences University of the Punjab Lahore Pakistan; ^2^ Turku Centre for Biotechnology University of Turku and Åbo Akademi University Finland; ^3^ Department of Microbiology College of Life Sciences Wuhan University Hubei Province China; ^4^ The Research Organization of Science and Technology Ritsumeikan University Kusatsu Shiga Japan

**Keywords:** crystal structure, thermophilicity, tryptophan biosynthesis, zinc binding

## Abstract

Anthranilate phosphoribosyltransferase (TrpD) is involved in tryptophan biosynthesis, catalyzing the transfer of a phosphoribosyl group to anthranilate, leading to the generation of phosphoribosyl anthranilate. TrpD belongs to the phosphoribosyltransferase (PRT) superfamily and is the only member of the structural class IV. X‐ray structures of TrpD from seven species have been solved to date. Here, functional and structural characterization of a recombinant TrpD from hyperthermophilic archaeon *Thermococcus kodakarensis* KOD1 (*Tk*TrpD) was carried out. Contrary to previously characterized Mg^2+^‐dependent TrpD enzymes, *Tk*TrpD was found to have a unique divalent cation dependency characterized by maximum activity in the presence of Zn^2+^ (1580 μmol·min^−1^·mg^−1^, the highest reported for any TrpD) followed by Ca^2+^ (948 μmol·min^−1^·mg^−1^) and Mg^2+^ (711 μmol·min^−1^·mg^−1^). *Tk*TrpD displayed an unusually low thermostability compared to other previously characterized proteins from *T. kodakarensis* KOD1. The crystal structure of *Tk*TrpD was determined in free form and in the presence of Zn^2+^ to 1.9 and 2.4 Å resolutions, respectively. *Tk*TrpD structure displayed the typical PRT fold similar to other class IV PRTs, with a small N‐terminal α‐helical domain and a larger C‐terminal α/β domain. Electron densities for Zn^2+^ were identified at the expected zinc‐binding motif, DE(217–218), of the enzyme in each subunit of the dimer. Two additional Zn^2+^ were found at a new dimer interface formed in the presence of Zn^2+^. A fifth Zn^2+^ was found bound to Glu118 at crystal lattice contacts and a sixth one was ligated with Glu235. Based on the *Tk*TrpD–Zn^2+^ structure, it is suggested that the formation of a new dimer may be responsible for the higher enzyme activity of *Tk*TrpD in the presence of Zn^2+^ ions.

**Abbreviations**
LB: Luria–Bertani
PRA: phosphoribosyl anthranilate
PRPP: phosphoribosyl pyrophosphate
PRTs: phosphoribosyltransferases
rmsd: root mean square deviation
TrpD: anthranilate phosphoribosyltransferase

Anthranilate phosphoribosyltransferase (TrpD, EC2.4.2.18) catalyzes the second step in tryptophan biosynthesis, which involves the transfer of a phosphoribosyl group to anthranilate to generate phosphoribosyl anthranilate (PRA), the basic skeleton of tryptophan (Fig. S1). TrpD belongs to the functional superfamily of phosphoribosyltransferases (PRTs) 1, which play important role in the metabolism of nucleotides and amino acids 2.

Phosphoribosyltransferases have been divided into four different classes on the basis of their tertiary structures 3, 4. Class I has a common α/β fold and comprises uracil, orotate, and purine PRTs. Class II has an N‐terminal α/β sandwich domain and a C‐terminal α/β TIM barrel domain. This class includes the quinolinate and nicotinic acid PRTs. Class III has a unique domain structure and includes ATP‐PRTase. Class IV PRTs are limited to TrpD 5 and exhibit a homodimeric structure and a novel PRT fold, consisting of a small N‐terminal α‐helical domain connected to a large C‐terminal α/β domain by a hinge region 6. The X‐ray structures of TrpD enzymes from *Sulfolobus solfataricus* (*Ss*TrpD; PDB entry 2GVQ) 7, *Pectobacterium carotovorum* (*Pc*TrpD; PDB entry 1KHD) 8, *Mycobacterium tuberculosis* (*Mtb*TrpD; PDB entry 4X5B) 9, *Thermus thermophilus* (*Tt*TrpD; PDB entry 1V8G; Shimizu and Kunishima, 2004, RIKEN Structural Genomics/Proteomics Initiative, unpublished), *Acinetobacter* sp. *ADP1* (*As*TrpD; PDB entry 4YI7; Evans *et al*., 2015, unpublished), *Xanthomonas campestris* (*Xc*TrpD; PDB entry 4HKM; Ghosh *et al*., 2012; New York Structural Genomics Research Consortium, unpublished), and *Nostoc* sp. (*Ns*TrpD; PDB entry 1VQU; Joint Center for Structural Genomics, 2005, unpublished) have been solved.

Most of PRTs have been shown to utilize Mg^2+^ as divalent cation for enzyme activity. However, *Salmonella typhimurium* and *P. carotovorum* TrpD enzymes have been found to utilize other metal ions, including Mn^2+^ and Co^2+^ in addition to Mg^2+^, for enzyme activity 8, 10. These divalent cations have been implicated in phosphoribosyl pyrophosphate (PRPP) complexation, which induces prominent ordering of a conserved Gly‐rich loop GTGGD in TrpD 7.

Here, we report the biochemical and structural characterization of TrpD (*Tk*TrpD) from the hyperthermophilic archaeon *Thermococcus kodakarensis* KOD1, an obligate heterotroph that grows optimally at 85 °C and pH 6.5 11. The gene encoding *Tk*TrpD was expressed in *Escherichia coli* and the recombinant gene product was purified, characterized, crystallized and its crystal structure was determined in free form as well as in the presence of Zn^2+^ to 1.9 and 2.4 Å resolutions, respectively. The results would provide a better understanding of the TrpD family of enzymes and help in biotechnological applications to synthesize compounds for use in biochemical assays 12, 13. Moreover, TrpD has also emerged as a potential candidate for biomedical applications. The importance of TrpD has been emphasized by a genome‐wide transposon mutagenesis study in *M. tuberculosis* 14, which showed that the enzymes responsible for the biosynthesis of PRPP as well as biosynthetic enzymes that use PRPP, such as TrpD, are essential for mycobacterial growth 14, 15.

## Results and Discussion

### Production and purification of *Tk*TrpD

**Figure 1:**
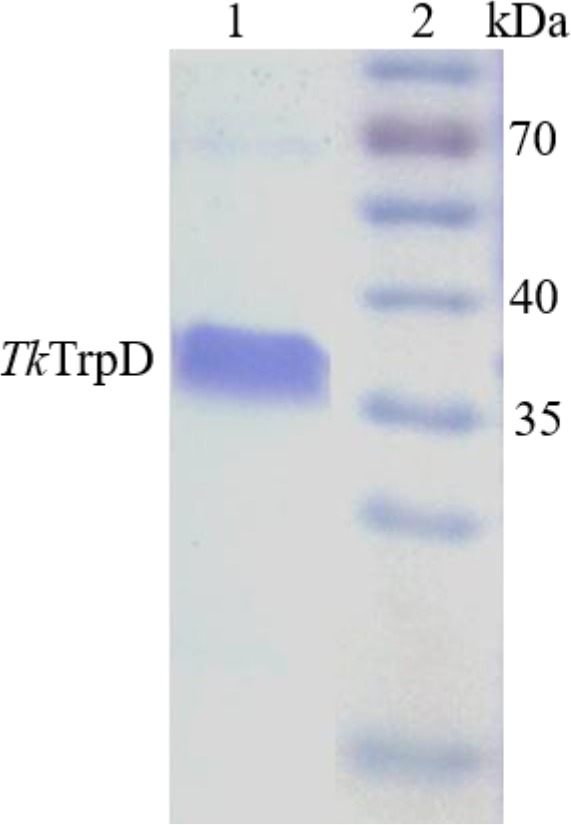
Purity of *Tk*TrpD in SDS/PAGE stained with Coomassie Brilliant Blue. Lane 1, Purified *Tk*TrpD eluted after ResQ column chromatography; Lane 2, molecular mass marker (Page Ruler prestained protein ladder # SM 0671, Fermentas).

The *Tk*TrpD gene (KEGG entry: TK0253) consists of an open reading frame (ORF) of 978 nucleotides, encoding for a polypeptide of 325 amino acid residues with a theoretical molecular mass of 34346.16 Da and pI of 4.9. *Tk*TrpD was produced in *E. coli* and purified to homogeneity using heat treatment and ion‐exchange chromatography. Purified recombinant *Tk*TrpD exhibited a molecular weight of about 36 kDa (Fig. 1), matching the molecular weight calculated from the amino acid sequence. By gel filtration chromatography, the molecular mass of *Tk*TrpD was estimated to be 70 kDa, indicating that *Tk*TrpD is a homodimer in solution (Fig. S2).

### Effect of pH and temperature

**Figure 2:**
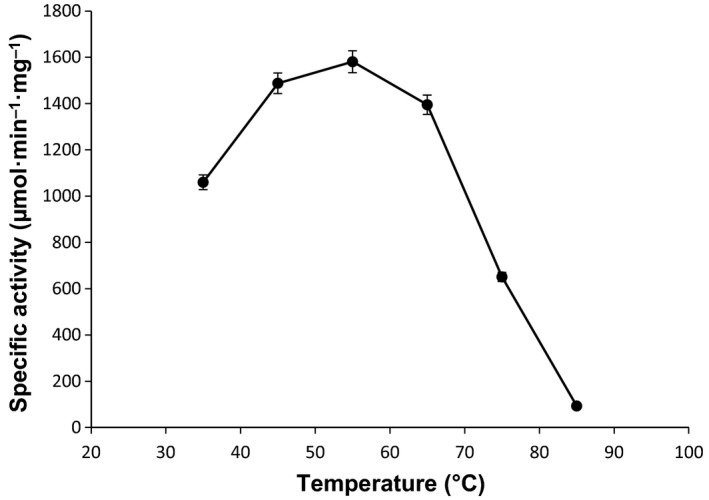
Optimal temperature for *Tk*TrpD enzymatic activity. The activity assays were conducted in triplicate at pH 8.5 and at various temperatures (35–85 °C).

The optimal pH for *Tk*TrpD activity was found to be 8.5–9.0 (Fig. S3). The effect of temperature on *Tk*TrpD activity was examined at optimal pH. *Tk*TrpD exhibited highest activity at 55 °C (Fig. 2) although the optimal growth temperature of *T. kodakarensis* is 85 °C. This result is in contrast to most of the enzymes from hyperthermophiles but similar to ribose‐5‐phosphate pyrophosphokinases from *T. kodakarensis* 16 and *Pyrobaculum calidifontis* 12, and phosphoribosyl diphosphate synthase from *S. solfataricus* 17. It should be noted that a protective mechanism of protein stabilization in hyperthermophiles has been suggested involving the secretion of small‐molecule osmolytes in stressful conditions 18. A similar mechanism may apply to *Tk*TrpD to increase its stability and prevent unfolding at elevated temperatures.

### Cation dependency

**Figure 3:**
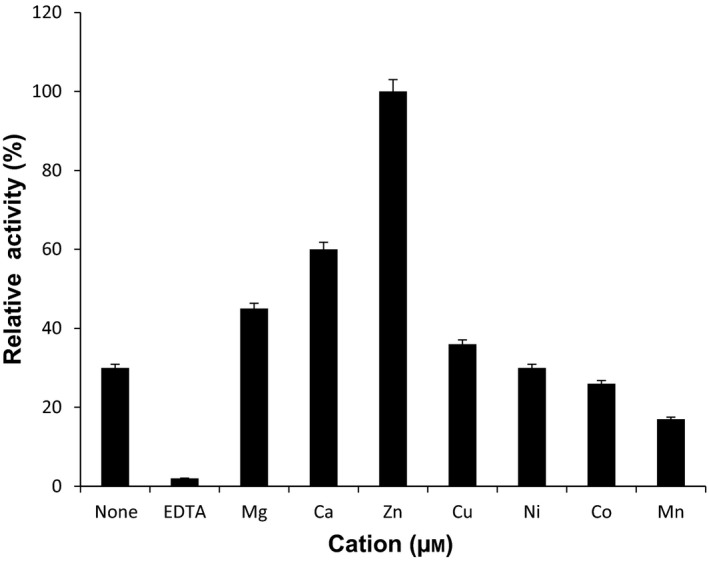
Effect of metal cations on *Tk*TrpD activity. Reactions were performed at pH 8.5 and temperature of 55 °C. Chloride salt of each cation and EDTA were used at 100 μm concentration. Each measurement is the average value of three independent experiments.

Anthranilate phosphoribosyltransferase enzymes from *E. coli*,* S. typhimurium*,* Saccharomyces cerevisiae*,* S. solfataricus,* and *M. tuberculosis* have been reported to be dependent on Mg^2+^ for enzymatic activity 15, 19, 20. *Pectobacterium carotovorum* and *S. typhimurium* TrpDs have been reported to be activated by Mn^2+^ 8, 10. Addition of EDTA completely inhibited the enzymatic activity of *Tk*TrpD, indicating the dependency of the enzyme on metal cations. The effect of various cations on *Tk*TrpD was therefore examined (Fig. 3). Surprisingly, addition of Zn^2+^ and Ca^2+^ led to higher specific activities than Mg^2+^, whereas Cu^2+^, Ni^2+^, Co^2+^, and Mn^2+^ showed lower activities. The decrease in enzyme activity in the presence of Co^2+^ and Mn^2+^ may be attributed to the slight precipitation of *Tk*TrpD in the presence of these metal ions.

### Effect of cation concentration

**Figure 4:**
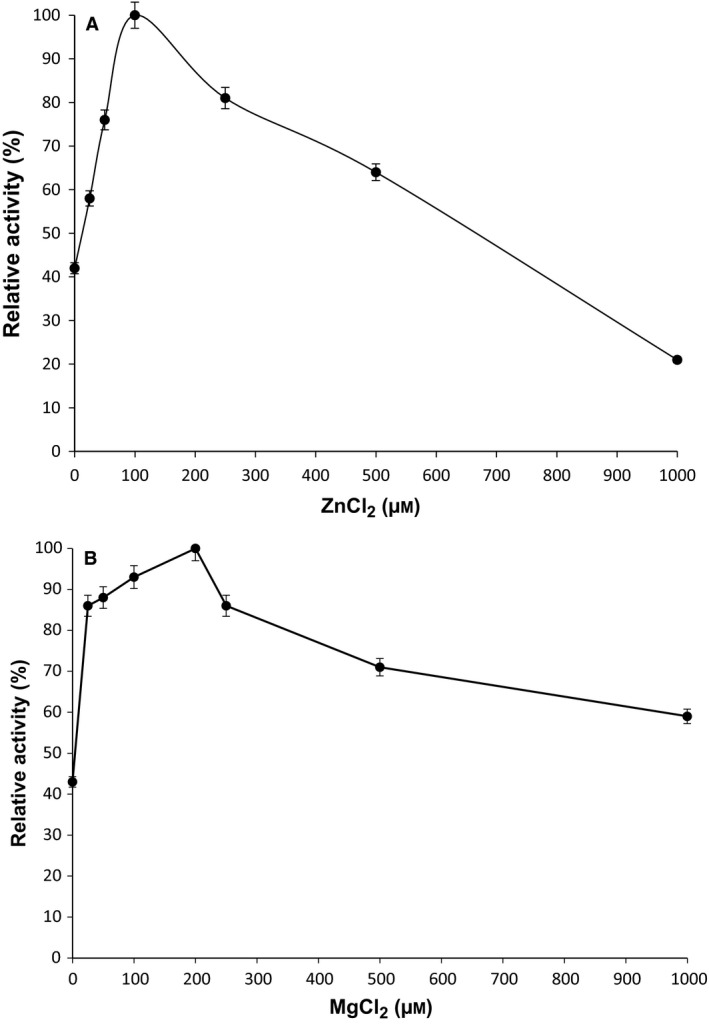
(A) Effect of ZnCl_2_ on *Tk*TrpD activity. The activity assays were conducted with various concentrations of ZnCl_2_ at pH 8.5 and temperature of 55 °C in triplicate. (B) Effect of MgCl_2_ on *Tk*TrpD enzyme activity. The activity assays were conducted with various concentrations of MgCl_2_ at pH 8.5 and temperature of 55 °C in triplicate.

*Tk*TrpD activity increased with the addition of Zn^2+^ until the Zn^2+^ concentration reached 100 μm. Higher concentrations of Zn^2+^ significantly inhibited the reaction (Fig. 4A). In the case of Mg^2+^, the activity was maximal at 200 μm; however, concentrations above 200 μm also decreased enzyme activity (Fig. 4B).

### Kinetic parameters

**Figure 5:**
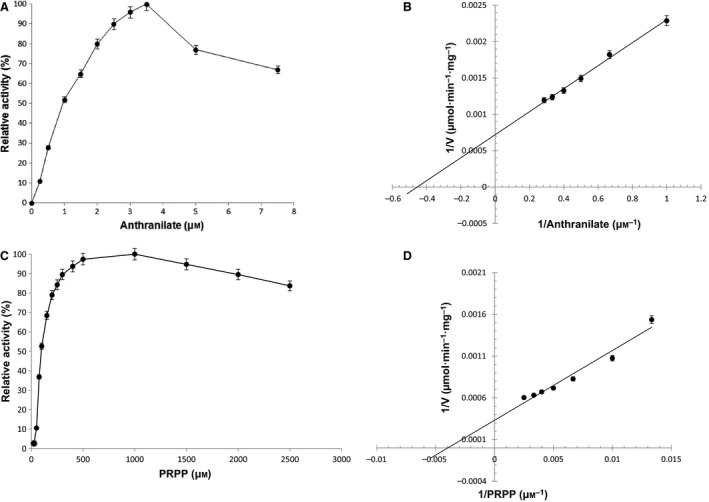
Effect of anthranilate and PRPP on *Tk*TrpD activity. (A) and (C) show relative activity, whereas (B) and (D) show Lineweaver–Burk plot of steady‐state kinetic analysis. The kinetic parameters were examined at temperature of 55 °C and pH 8.5, in the presence of 100 μm ZnCl_2_ in triplicate.

The effect of substrate concentration on *Tk*TrpD activity was investigated in the presence of Zn^2+^. Anthranilate and PRPP were the two substrates used in the assays. The first substrate, PRPP, was kept constant at 1 mm during the measurement of the kinetic parameters toward anthranilate. Similarly, the second substrate, anthranilate, was kept constant at 4 μm when the kinetic parameters toward PRPP were measured. Anthranilate concentrations above 4 μm resulted in reduced enzymatic activity, suggesting substrate inhibition by anthranilate as observed also in *M. tuberculosis* TrpD 21. Apparent *K*_m_ values for anthranilate and PRPP were 2.2 μm and 250 μm, respectively (Fig. 5). *Tk*TrpD was highly active with specific activity of 1580 μmol·min^−1^·mg^−1^. To the best of our knowledge, this is the highest enzyme activity for any TrpD reported so far. A comparison of kinetic parameters and specific activities of characterized TrpDs from various sources is shown in Table 1.

**Table 1:** Comparison of kinetic parameters of TrpD from various organisms. ND: no data available

Organisms	*K*_m_ anthranilate (μm)	*K*_m_ PRPP (μm)	Specific activity (μmol^·^min^−1·^mg^−1^)	Reference
*Thermococcus kodakarensis*	2.2	250	1580	This study
*Sulfolobus solfataricus*	0.085	180	ND	2
*Escherichia coli*	0.28	50	ND	39
*Salmonella typhimurium*	5.9	3.8	1350	40
*Pectobacterium carotovorum*	ND	ND	24.5	41
*Serratia marcescens*	ND	ND	409	42
*Aerobacter aerogenes*	ND	ND	734	43
*Neurospora crassa*	ND	ND	16	44
*Salmonella enterica* subsp. *enterica* serovar *typhimurium*	ND	ND	1.54	45
*Saccharomyces cerevisiae*	1.6	22.4	1.58	19
*Hansenula henricii*	4.6	880	0.4	46
*Corynebacterium glutamicum*	ND	ND	0.049	47

### Quality of the *Tk*TrpD structure

**Figure 6:**
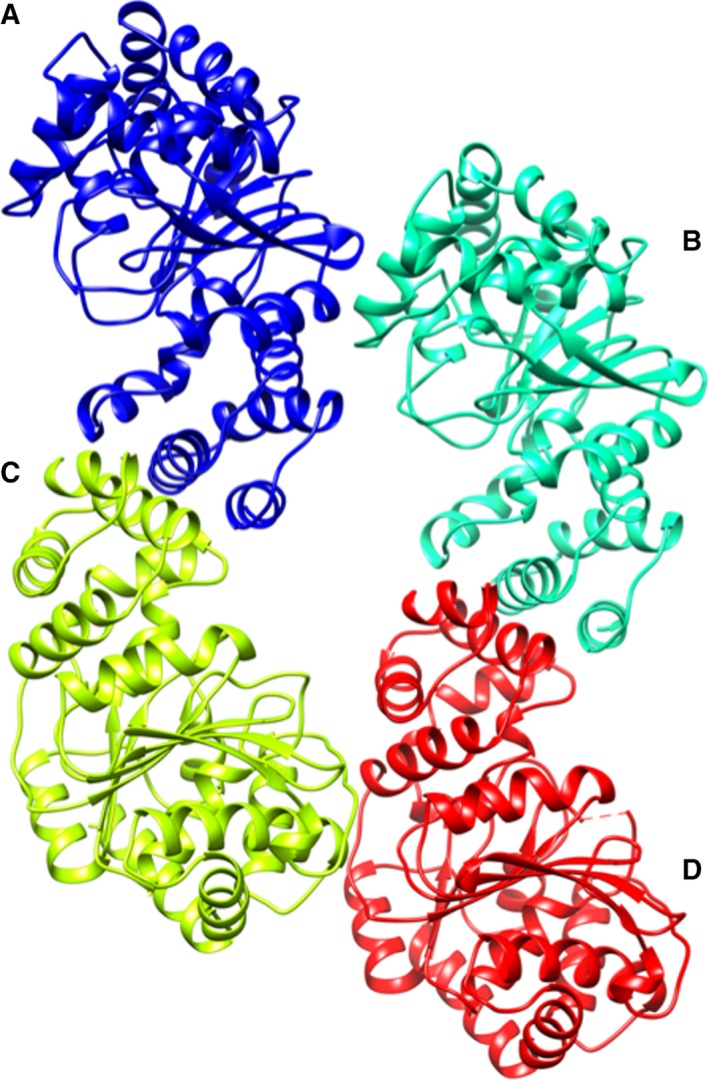
Ribbon diagram of *Tk*TrpD tetramer in the asymmetric unit. Each subunit of the tetramer is shown in different color with the functional homodimers formed between A–C and B–D. Figure was created using UCSF Chimera 50.

*Tk*TrpD crystallizes with four molecules (A, B, C, and D) in the asymmetric unit that form two homodimers (A–C and B–D) (Fig. 6). The Matthews coefficient *V*_M_ 22 for four molecules in the asymmetric unit is 2.4 Å^3·^Da^−1^, corresponding to a solvent content of ∼48.5%. The refined structure shows a root mean square deviation (rmsd) of 0.012 Å and 1.15° from the ideal values of bond lengths and angles, respectively. The observed crystal form of *Tk*TrpD soaked with ZnCl_2_ has a dimer (A, B) in the asymmetric unit. The Matthews coefficient *V*_M_ for two molecules in the asymmetric unit is 2.3 Å^3^·Da^−1^, corresponding to a solvent content of ~ 46.2%. As the crystals used for the Zn^2+^ soaking had been grown in different conditions, unsoaked crystals were tested and found to have similar space group and cell dimensions as those of the free *Tk*TrpD, suggesting that soaking with Zn^2+^ induced a rearrangement of the crystal packing. The refined structure shows an rmsd of 0.010 Å and 1.41° from the ideal values of bond lengths and bond angles, respectively. Detailed statistics of data collection and refinement for both structures are presented in Table 2.

**Table 2:** *Tk*TrpD data collection and refinement statistics^a^

Data collection	Free *Tk*TrpD	*Tk*TrpD‐Zn^2+^
Wavelength (Å)	0.96598	1.03320
Beamline	MASSIF‐1, ESRF	P13, PETRA III
Detector	PILATUS 2M	PILATUS 6M
Temperature (K)	100	100
Space group	*P*2_1_2_1_2_1_	*P*22_1_2_1_
Unit cell, *a*,* b*,* c* (Å)	83.9, 85.6, 180.8	42.6, 81.3, 179.4
α, β, γ (°)	90.0, 90.0, 90.0	90.0, 90.0, 90.0
Mosaicity (°)	0.05	0.19
Resolution range (Å)	49.27–1.91 (1.98–1.91)	48.19–2.42 (2.50–2.42)
Total no. of measurements	453 345	172 488
No. of unique reflections	100 253	24 674
Completeness (%)	98.8 (95.9)	99.8 (99.0)
Multiplicity	4.5 (4.6)	7.0 (7.2)
<*I*/σ (*I*)>	9.5 (1.5)	9.6 (1.4)
*R*_meas_ 48 (%)	10.2 (113)	17.2 (152)
CC_1/2_ 49	0.997 (0.536)	0.997 (0.467)
Overall B factor from Wilson plot (Å^2^)	28.9	59.0
Refinement		
Resolution	49.27–1.91 (1.98–1.91)	48.19–2.42 (2.50–2.42)
No. of reflections (working/test)	95 240/4930	23 378/1230
*R*_cryst_/*R*_free_ (%)	18.6/23.5	20.4/25.3
No. of protein atoms	9635	4835
No. of water molecules	971	127
No. of protein ligands	–	9 (6Zn^2+^, 2Na^+^, 1Cl^−^)
rmsd in bond lengths (Å)	0.012	0.010
rmsd in bond angles (°)	1.16	1.41
Residues in most favorable regions (%)	96.0	96.0
Residues in additionally allowed regions (%)	3.3	3.9
Average B factor (Å^2^)		
Protein	35.9	52.6
Water	41.9	52.4
Ligands	–	66.3
PDB id	5NOE	5NOF

a Values in parentheses are for the outer resolution shell.

### Overall structure

**Figure 7:**
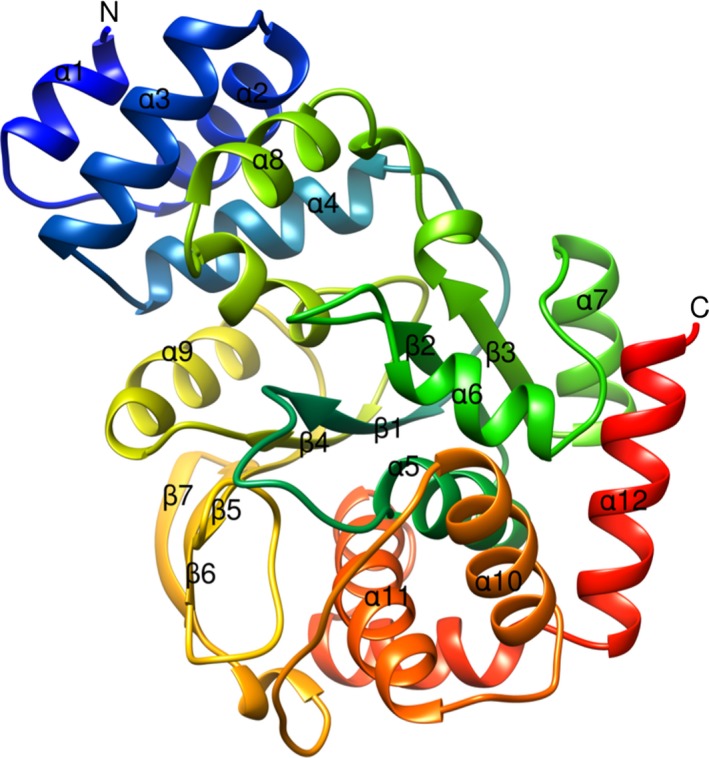
Ribbon diagram of X‐ray crystal structure of *Tk*TrpD. Only one subunit of the homodimer is shown, with the amino acid chain colored from blue at the N terminus to red at the C terminus. Each subunit consists of a small α‐helical domain containing four helices (α1, α2, α3, and α4) and a larger C‐terminal α/β domain with a central β‐sheet containing seven β‐strands (six parallel and one antiparallel) surrounded by eight α‐helices. Figure was created using UCSF Chimera.

*Tk*TrpD structure displays the PRT fold similar to other PRTs. Each *Tk*TrpD molecule consists of 325 residues arranged in two domains (Fig. 7): a small N‐terminal α‐helical domain containing four helices and a large C‐terminal α/β domain with a central sheet of seven β‐strands (six parallel and one antiparallel) surrounded by eight α‐helices. A hinge region (α4‐β1, β3‐α8, and α9–β4) connects the two domains.

The N‐terminal domain is involved in dimer formation in TrpD enzymes (*Ss*TrpD, *Mtb*TrpD, *Tt*TrpD, *As*TrpD, *Xc*TrpD, *Ns*TrpD, and *Pc*TrpD). Similarly, *Tk*TrpD subunits also associate with each other at their N‐terminal ends through their small α‐helical domains (C‐terminal end of α1, α3, and α8). In *Ss*TrpD, residues Ile36 and Met47 have been shown to be involved in dimerization. Mutations of these residues resulted in loss of dimeric form with decreased thermal stability 2. Both of these residues are not conserved in TrpD family. The corresponding residues in *Tk*TrpD are Val31 and Thr42. Analysis of protein–protein interactions with PDBsum 23 shows that Ala35, Thr42 (located at the N and C termini of α3, respectively) and Leu162 (C‐terminal end of α8) are found to form highest number of inter‐subunit interactions, showing that mostly hydrophobic residues are involved in inter‐subunit interactions in *Tk*TrpD dimer formation in agreement with dimer formation in other TrpD enzymes.

### Structural comparison

**Figure 8:**
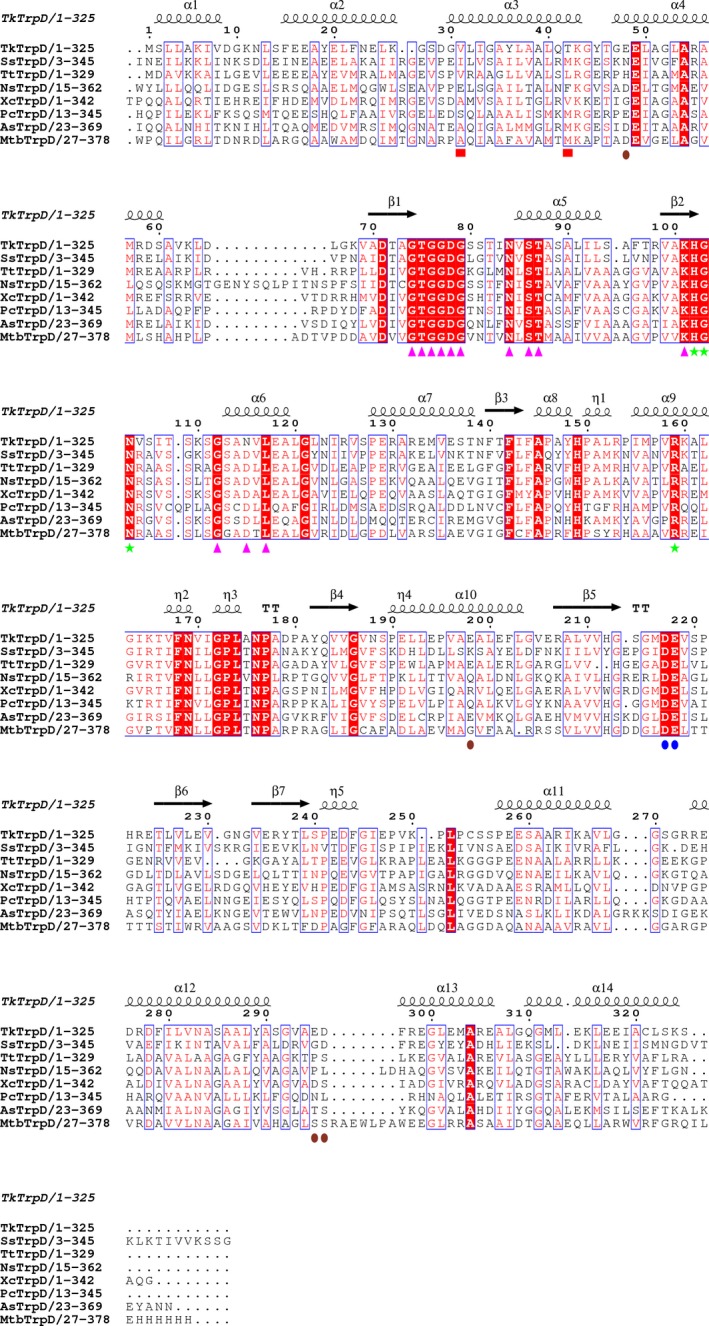
Structure‐based sequence alignment of *Tk*TrpD. The enzymes used are *Ss*TrpD (PDB entry 2GVQ) 7, *Tt*TrpD (PDB entry 1V8G; Shimizu *et al*., 2004, unpublished), *Ns*TrpD (PDB entry 1VQU; Joint Center for Structural Genomics, 2005, unpublished), *Xc*TrpD (PDB entry 4HKM; Ghosh *et al*., 2012, unpublished), *Pc*TrpD (PDB entry 1KHD) 8, *As*TrpD (PDB entry 4YI7; Evans *et al*., 2015, unpublished), and *Mtb*TrpD (PDB entry 4X5B) 9. Figure was created using espript 3.0 51. Conserved residues are indicated by white letters on a red background (strictly conserved) or red letters on a white background (global similarity score, 0.7) and framed in blue boxes. Markers indicate residues postulated to be involved in PRPP binding (magenta arrows), anthranilate binding (green stars), metal binding (blue ovals), and dimerization (red boxes). Residues involved in Zn^2+^ binding at the TrpD–Zn^2+^ dimer interface are shown with brown ovals.

Structure‐based sequence alignment of *Tk*TrpD (Fig. 8) shows highest homology with *Ss*TrpD (45%) followed by *Tt*TrpD (43%), *Ns*TrpD (39%), *Xc*TrpD (39%), *Pc*TrpD (37%), *As*TrpD (36%), and *Mtb*TrpD (34%). Several conserved sequences were found that play an important role in catalysis. When superimposed with *Ss*TrpD, *Tk*TrpD shows rmsd of 1.15 Å for 256 Cα‐atoms. At the C‐terminal end of *Ss*TrpD, there is a small helix of 10 residues which is absent in *Tk*TrpD and all other reported TrpD structures.

### Active site

Each monomer has an active site in a cleft found in the hinge region. In *Tk*TrpD, substrate (anthranilate + PRPP) binding positions were found conserved as in other TrpDs. A conserved anthranilate binding motif (KHGN(101–104)) was found in β2–α6 loop. Lys101, in particular, is involved in anthranilate binding and HGN(102–104) in PRPP binding. This motif has been determined to be involved in catalysis in previously determined TrpD structures (e.g., *Ss*TrpD, *Mtb*TrpD). Arg159 in *Tk*TrpD found on helix α8 is also conserved and in previous structures 7, 15 has been shown to be involved in anthranilate binding by forming hydrogen bond to it and is essential for catalytic function. The corresponding residues in *Mtb*TrpD and *Ss*TrpD are Arg193 and Arg164, respectively. A highly conserved Gly‐rich sequence GTGGD(74–78) found in *Tk*TrpD in β1–α5 loop is considered as a signature motif of TrpD family and is involved in PRPP binding. Identical sequences have been found in *Mtb*TrpD (GTGGD(107–111)) and in *Ss*TrpD (GTGGD(79–83)). The first Gly of this region, in particular, is known to interact with the PPi group of PRPP via its peptide amino group and also with the amino group of anthranilate.

### Divalent ion binding sites

Metal ions bind to two sites in the TrpD family. The first metal ion binds to pyrophosphate and ribose oxygen atoms of PRPP, and this site is common in PRT superfamily. The second site is specific for TrpD family and involves a conserved DE motif whose residues are key to metal binding and are invariant in all TrpD enzymes structurally characterized until now (Table 3).

**Table 3:** Divalent ion binding sites in TrpD family

Ions	Contacts	Reference
*Mtb*TrpD		
Mg^2+^‐I	S119, E252, and PRPP	15
Mg^2+^‐II	DE(251–252)	
*Ss*TrpD		
Mg^2+^‐I	OH^−^ groups of ribose and pyrophosphate oxygens of PRPP	7
Mg^2+^‐II	DE(223–224)	
*Pc*TrpD		
Mn^2+^‐I	S103, E237, and PRPP	8
Mn^2+^‐II	DE(236–237)	
*Tk*TrpD		
Zn^2+^‐I	DE(217–218), D78 (subunit A)	This study
Zn^2+^‐II	E118 (subunit A at crystal lattice contacts with E118 from a symmetry molecule)	
Zn^2+^‐III	E235 (subunit B)	
Zn^2+^‐IV	DE(217–218), D78 (subunit B)	
Zn^2+^‐V	E198‐A and E295‐B	
Zn^2+^‐VI	ED(295–296)‐B, E48‐A, E198‐A	

**Figure 9:**
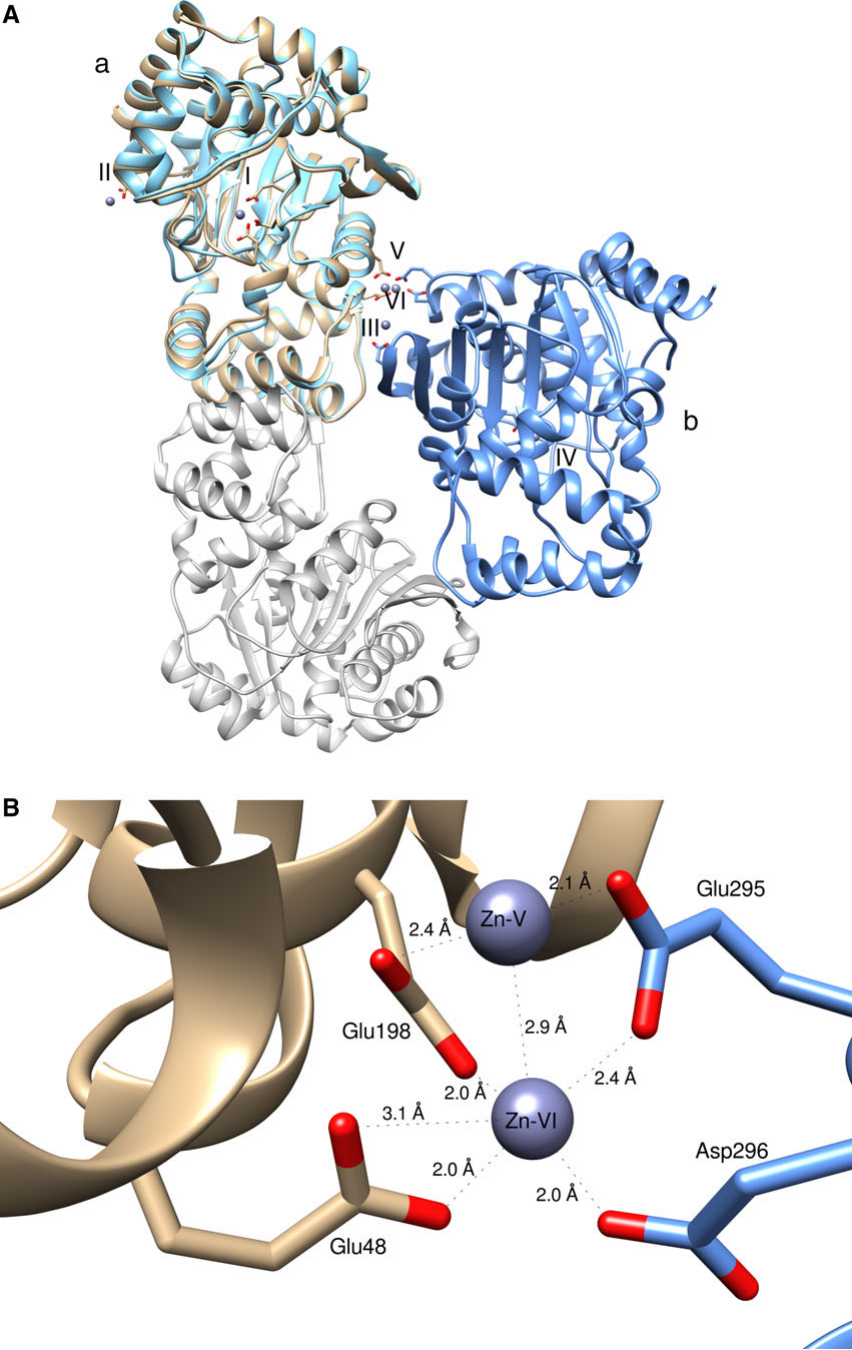
(A) Superposition of *Tk*TrpD dimers with and without Zn^2+^. Subunit (a) is shown in brown (with Zn^2+^) and cyan (without Zn^2+^). The Zn^2+^‐binding sites are labeled with latin numbers as in the text. The second subunit of the dimer is colored in gray (without Zn^2+^, it corresponds to C as in Fig. 6) and in blue (with Zn^2+^) (B) The interface of *Tk*TrpD with the bound Zn^2+^ ions. The subunits are colored differently. Zn^2+^ ions are shown as dark slate blue spheres. Distances are depicted. Figures were created using UCSF Chimera.

Soaking of *Tk*TrpD with a ZnCl_2_ solution resulted in the identification of a total of six Zn^2+^ ions in the dimer, while in other TrpDs only four Zn^2+^ ions per dimer are present. In each subunit of *Tk*TrpD, one Zn^2+^ ion was found in the primary metal binding site, involving the conserved DE(217–218) motif and Asp78. As PRPP is not present in the structure, no additional Zn^2+^ ion was found in the vicinity of the primary metal binding site. The structure of *Tk*TrpD soaked with zinc has a dimer in the asymmetric unit. Gel filtration has also shown that in the presence of Zn^2+^, *Tk*TrpD exists as a dimer in solution (Fig. S2). Structural comparison of the Zn^2+^‐free and Zn^2+^‐bound structures at subunit level shows low rmsd between Cα atoms (0.63 Å), suggesting no significant changes. Notable changes, however, were identified in the position of helices α8 and α9 that move toward the active site in the Zn^2+^‐bound structure. Most importantly, following superposition with the Zn^2+^‐free *Tk*TrpD (Fig. 9A), the structure solution of the Zn^2+^‐bound *Tk*TrpD revealed a different arrangement of the two subunits compared to the typical dimer found in other TrpD enzymes. Interestingly, two Zn^2+^ ions (V and VI) were found at the interface between Glu48 and Glu198 of subunit A and ED(295–296) of subunit B in the dimer. The two zinc ions are close to each other with a distance of 2.9 Å (Fig. 9B) and have similar B‐factors (43.7 and 46.0 Å^2^, respectively). These two additional Zn^2+^ binding sites in *Tk*TrpD may therefore explain the effect of Zn^2+^ on *Tk*TrpD by promoting a different dimer formation. At present, we cannot conclude whether this property is shared by this enzyme from other sources as well, or whether it is a unique property of *Tk*TrpD. However, the structure‐based alignment (Fig. 8) shows that the ED(295–296) motif is not conserved, and therefore, other TrpDs may be unable to adopt the same dimer arrangement. Sequence variations are also evident for Glu48 and Glu198.

In conclusion, the biochemical and structural characterization of *Tk*TrpD reported here may lead to new strategies to alter TrpD enzymatic activity. The new subunit–subunit interface may play a role in the increased activity of *Tk*TrpD in the presence of Zn^2+^. For example, Glu198 belongs to helix α10 and slight alterations upon Zn^2+^ binding and dimer rearrangement could be traversed to the active site through the α8 and α9 helices. Alternatively, formation of the new dimer may affect the position of helix α8, which in the typical TrpD dimer is part of the conventional interface. In the new dimer, helix α8 becomes free from any interactions with a neighboring subunit, and therefore, it may be able to adopt more favorable positions for substrate binding. Further studies, however, are needed to elucidate the precise role of the Zn^2+^‐binding sites and their potential direct and indirect effects on the active site of the enzyme.

## Materials and methods

Chemicals and materials used in this study were purchased from either Thermo‐Fisher Scientific (Leicestershire, UK), Fluka (Buchs, Switzerland), Merck (Darmstadt, Germany), or Sigma‐Aldrich (St. Louis, MO, USA). Gene‐specific primers were commercially synthesized by Macrogen Inc (Seoul, Korea). *Escherichia coli* strains used were DH5α and BL21 Codon Plus (DE3)‐RIL (Stratagene, La Jolla, CA, USA). Luria–Bertani (LB) medium was used for the cultivation of *E. coli* strains.

### Gene cloning

Gene encoding *Tk*TrpD was amplified from genomic DNA of *T. kodakarensis*, using sequence‐specific forward F*Tk*TrpD (**CATATG**AGCCTTCTTGCGAAGATCGTCGATGG), which include a *Nde*I recognition site (shown in boldface) and reverse R*Tk*TrpD (TCAGCTTTTTGAGAGGCATGCTATCTCCTC) primers. PCR‐amplified gene product was ligated to cloning vector pTZ57R/T. The resultant recombinant plasmid PTZ‐*Tk*TrpD was digested with *Nde*I and *Hin*dIII to liberate *Tk*TrpD, which was cloned into the expression vector pET21a(+) using the same restriction sites. pET‐*Tk*TrpD name was assigned to the resultant recombinant expression plasmid. Presence of *Tk*TrpD in the expression plasmid was subsequently confirmed by restriction analysis and DNA sequencing.

### Gene expression and protein purification

*Escherichia coli* BL21 CodonPlus (DE3)‐RIL cells were transformed with recombinant pET‐*Tk*TrpD. Expression of gene was induced by 0.5 mm isopropyl‐β‐d‐thiogalactoside (IPTG). After induction for 6 h, cells grown in LB medium were harvested and resuspended in 50 mm Tris/HCl pH 8.5 buffer containing 1 mm DTT, 1 mm PMSF, and 20% v/v glycerol. For purification, soluble portion obtained after sonication was heat‐treated at 65 °C for 25 min and centrifuged (15 000 ***g*** for 15 min). ÄKTA Purifier chromatography system (GE Healthcare, Uppsala, Sweden) was used for further purification. Heat‐treated supernatant was applied to anion‐exchange QFF (6 mL) column (GE Healthcare) and the recombinant *Tk*TrpD was eluted with a linear gradient of 0–1 m NaCl. Fractions containing *Tk*TrpD were desalted by dialysis against 50 mm Tris/HCl (pH 8.5) buffer containing 1 mm DTT, 1 mm PMSF, and 20% v/v glycerol. Dialyzed *Tk*TrpD samples were applied to Resource Q (1 mL) column (GE Healthcare), and the protein was eluted with a linear gradient of 0–1 m NaCl. Analysis of the purified *Tk*TrpD was performed by SDS/PAGE. Molecular weight and oligomeric nature of *Tk*TrpD were determined by gel filtration chromatography column Superdex 75 10/300 GL attached to ÄKTA purifier (GE Healthcare). The standard curve was obtained with bovine pancreas chymotrypsinogen A (25 kDa), chicken egg white ovalbumin (48 kDa), and bovine serum albumin (63 kDa). Their gel‐phase distribution coefficient (*K*_av_) values were calculated and plotted against the log of their molecular weight (Fig. S3). Protein concentration was determined spectrophotometrically at every step of purification by Bradford reagent 24.

### Enzyme assays

*Tk*TrpD activity was determined fluorometrically by measuring the decrease in the concentration of anthranilic acid. Anthranilic acid reacts with PRPP, leading to the production of PRA (Fig. S1). The initial rate of decrease in anthranilate was measured, as anthranilate is utilized by *Tk*TrpD to form PRA, resulting in a decrease in emission/fluorescence at 390 nm. The activation and emission wavelengths for anthranilate were 315 and 390 nm, respectively. A standard curve was then used to convert fluorescent intensity to anthranilate concentration. The reaction mixture contained 4 μm anthranilate, 1 mm PRPP, 100 μm ZnCl_2_, 100 mm Tris/HCl buffer (pH 8.5), and 5 μg of *Tk*TrpD. The reaction mixture without PRPP was incubated at 55 °C for 5 min. The reaction started by adding PRPP at 55 °C and continued for 2.5 min. Two control experiments were carried out: one without enzyme and one without PRPP. The half‐life of PRPP is 56 min at 60 °C 20, suggesting that at 55 °C and for the time used for the reaction, no significant hydrolysis of PRPP is expected.

### Effect of temperature, pH, and metal ions

For the measurement of optimal temperature, enzyme assays were performed at various temperatures ranging from 35 to 85 °C keeping the pH constant. For the estimation of optimal pH, assays were performed at various pH values keeping the temperature unchanged at 55 °C. The following buffers were used: Na‐phosphate (pH: 6.0–7.0), Tris/HCl (pH: 7.0–9.0), and Na‐bicarbonate (pH: 9.0–10.0). The effect of divalent metal ions on the enzyme activity was investigated in the presence of either 50 or 100 μm of ZnCl_2_, MgCl_2_, CaCl_2_, MnCl_2_, NiCl_2_, CoCl_2_, and CuCl_2_. In case of EDTA, the final EDTA concentration was 100 and 2.5 mm. The effect of Zn^2+^ and Mg^2+^ concentration on the enzyme activity was measured in the range of 0–1 mm.

### Crystallization

Purified *Tk*TrpD was concentrated to 12 mg·mL^−1^ in 10 mm Tris/HCl (pH 8.0) buffer containing 0.1 m NaCl and 0.002% (w/v) NaN_3_. PACT screen (Molecular Dimensions, Suffolk, UK) was performed in 96‐well plate using the sitting drop vapor diffusion method. Promising crystals found in solution 79 (0.2 m sodium acetate (pH 7.5), 20% (w/v) PEG 3350) were optimized by the hanging‐drop vapor diffusion method at 16 °C in Linbro 24‐well cell culture plates. The reservoir solution consisted of 0.6 mL of condition 79 mixed with 0.2 mL of MilliQ water and the drops comprised 2 μL of 12 mg·mL^−1^ *Tk*TrpD mixed with 2 μL of reservoir solution. Crystals appeared after 1 day and were harvested after ~ 4 weeks for X‐ray data collection. Crystals were transferred to a reservoir solution supplemented with 20% v/v glycerol and flash‐cooled in liquid N_2_.

### ZnCl_2_ crystal soaking

*Tk*TrpD crystals obtained from solution 89 of the PACT screen (0.2 m sodium nitrate, 0.1 m Bistris‐propane buffer (pH 8.5), 20% (w/v) PEG 3350) were used after soaking in 100 mm ZnCl_2_ for ~ 5–10 min. The crystals were subsequently transferred to a reservoir solution supplemented with 23% v/v glycerol and flash‐cooled in liquid N_2_.

### Data collection and structure determination

Diffraction data for the free *Tk*TrpD were collected at ESRF (Grenoble) on the fully automatic high‐throughput MASSIF‐1 beamline 25 from a crystal that diffracted to 1.9 Å. xds 26 was used to index and integrate the data and aimless 27 for merging and scaling. The crystal was found to belong to the *P*2_1_2_1_2_1_ space group. *Ss*TrpD (PDB entry 2GVQ) 7 was found to be the best matched search model by molrep 28 as implemented in mrbump 29 from CCP4 30 and was used to obtain initial phases. After the solution was found, buccaneer 31 was employed for initial model building and automatic refinement with refmac5 32. Further refinement was carried out using phenix and water molecules were added with tools in phenix 33. Manual rebuilding and structure visualization was performed by coot 34. The progress of refinement was monitored using the *R*_free_ 35 with 5% of the reflections set aside.

#### ZnCl_2_ soaking

Data from a crystal soaked with ZnCl_2_ were collected at EMBL Hamburg (c/o DESY, Hamburg, Germany) on the P13 beamline at PETRA III and processed as before. Chain A of free *Tk*TrpD crystal structure was used as search model in phaser for structure determination by molecular replacement. The best solution was found in the orthorhombic *P*22_1_2_1_ space group. Refinement was initially carried out using phenix and water molecules were added with tools in phenix. At the final stages of refinement, PDB_REDO 36 was employed and refmac 32 was used. Manual rebuilding and structure visualization was performed by coot 34. The progress of refinement was monitored with the *R*_free_.

### Structure analysis

Interfaces were analyzed by PDBePISA 37. Structural superpositions were performed with PDBeFold 38 as implemented in coot 34. The superimposed structures were visually inspected using coot.

## Data Accessibility

Structural data are available in the Protein Data Bank under the accession numbers 5NOE and 5NOF.

## Author contributions

SP performed experiments, analyzed data, and wrote the manuscript. NR analyzed data, planned experiments, and wrote the manuscript. XFT performed experiments and analyzed data. TI planned experiments and analyzed data. ACP performed and planned experiments, analyzed data, and wrote the manuscript.

## Supporting information


**Fig. S1**. Reaction catalyzed by TrpD.
**Fig. S2**. Gel filtration elution profile of *Tk*TrpD with and without Zn^2+^.
**Fig. S3**. Determination of optimal pH for *Tk*TrpD enzymatic activity.
