# Supplementary material for: Anthranilate phosphoribosyltransferase from the hyperthermophilic archaeon Thermococcus kodakarensis shows maximum activity with zinc and forms a unique dimeric structure
Source: FEBS Open Bio. 2017 Jul 24;7(8):1217–30. doi: 10.1002/2211-5463.12264 (PMC5537072; doi:10.1002/2211-5463.12264)
Supplement: Supplementary file 1 — Fig. S1. Reaction catalyzed by TrpD. Fig. S2. Gel filtration elution profile of TkTrpD with and without Zn2+. Fig. S3. Determination of optimal pH for TkTrpD enzymatic activity. [file FEB4-7-1217-s001.pdf]

Supplementary material

Figure S1 Reaction catalyzed by TrpD

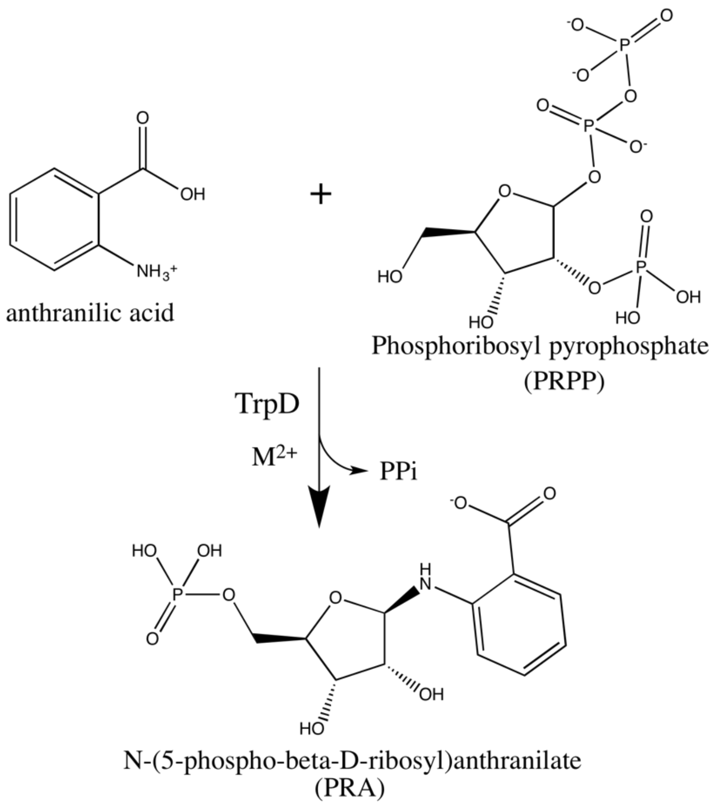

Figure S2 Elution profile of *Tk*TrpD from Superdex 75 10/300 gel filtration column. The retention volume (RV) is depicted on the X-axis. The buffer was 50 mM Tris-HCl, 150 mM NaCl, pH 8 and the flowrate 0.2 mL/min.

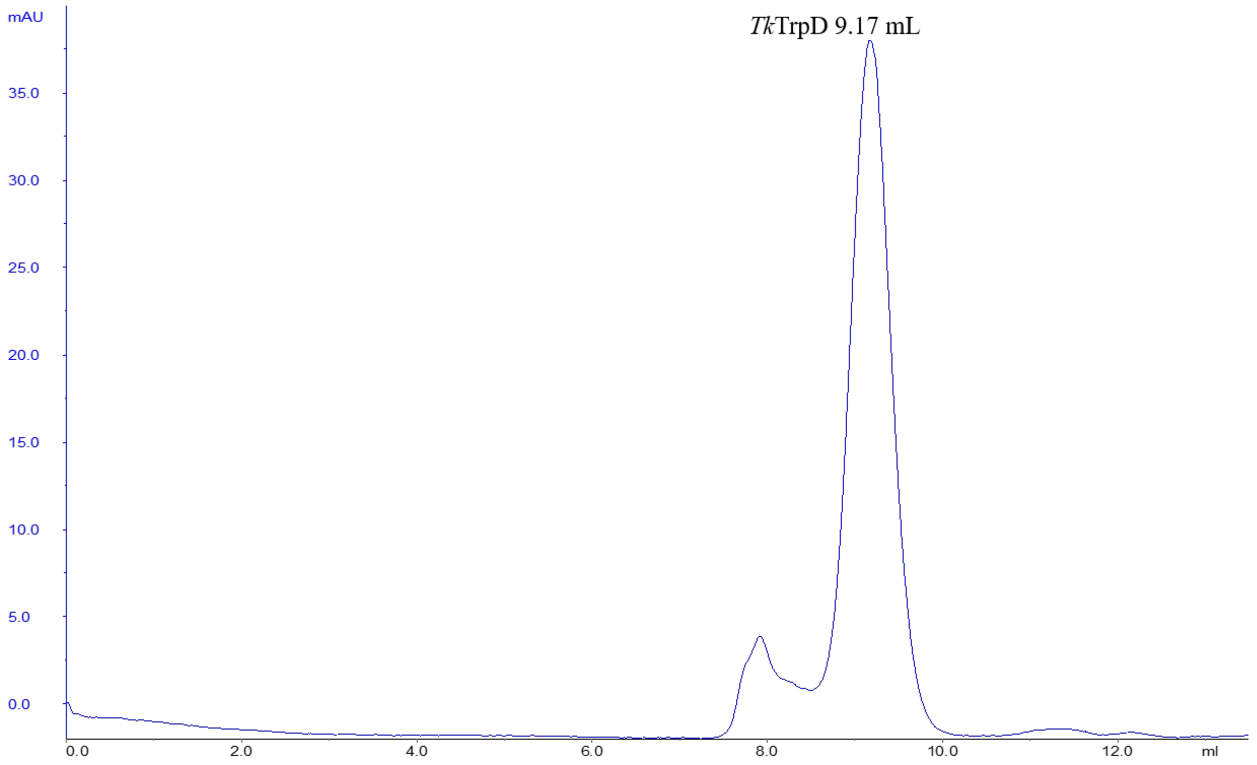

Gel-filtration chromatograph for *Tk*TrpD. The chromatograph shows the protein peaks observed as A280 versus the retention volume.

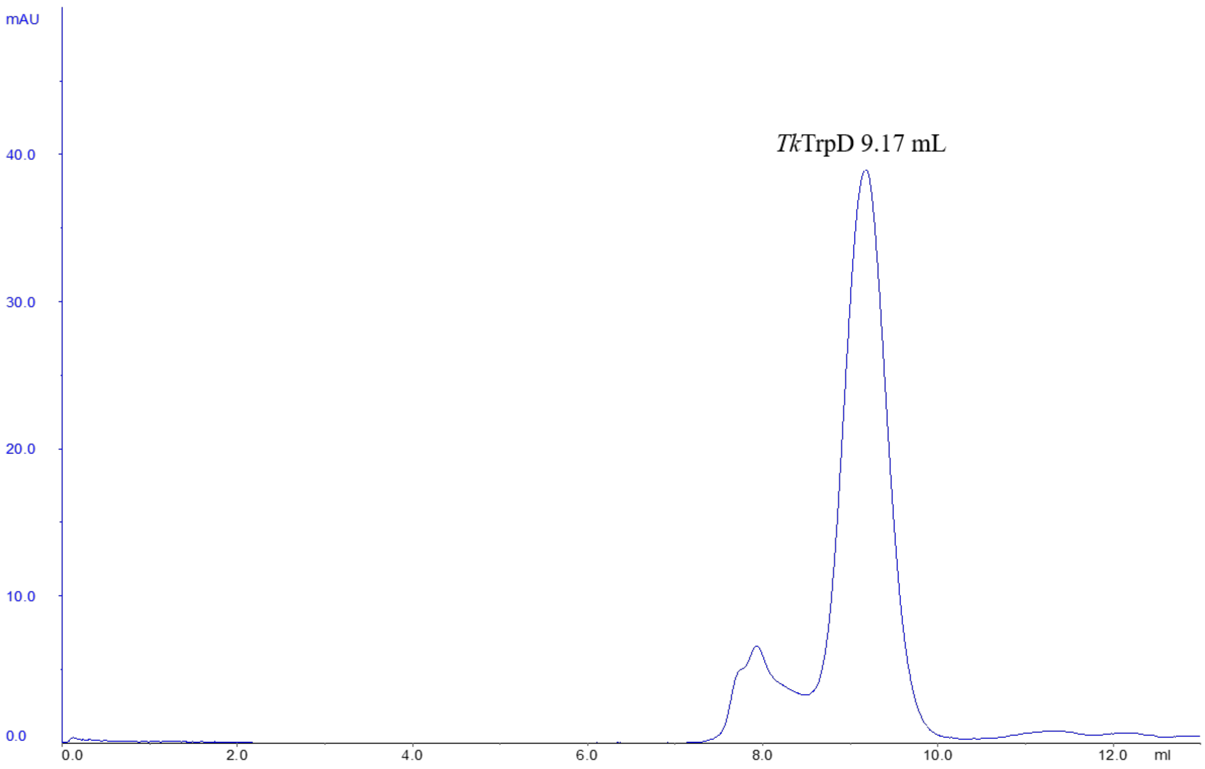

Gel-filtration chromatograph for *Tk*TrpD in the presence of Zn^{2+}. The chromatograph shows the protein peaks observed as A280 versus the retention volume.

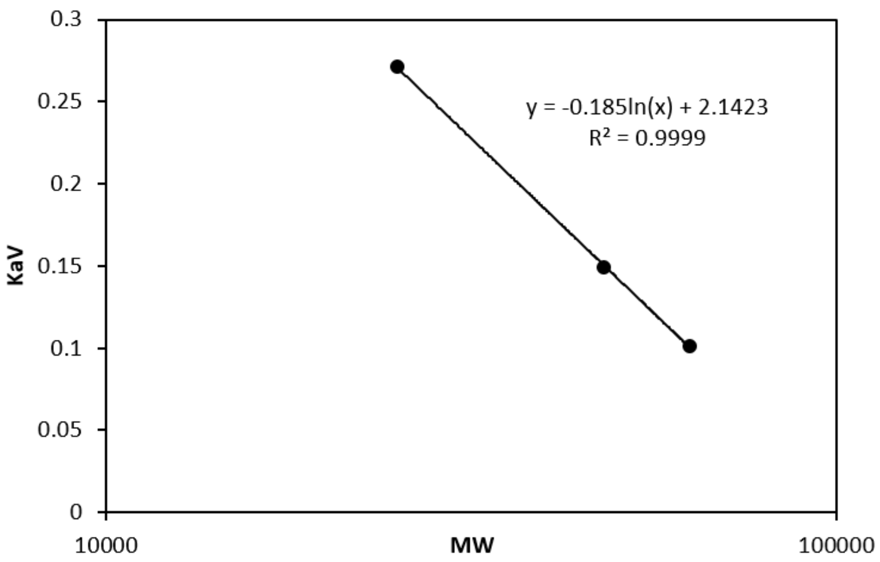

Standard curve for molecular weight determination of *Tk*TrpD by gel filtration. The gel phase distribution coefficient (K_{av}) values for standard proteins are plotted against log of their molecular weight.

Figure S3 Optimal pH for *Tk*TrpD enzymatic activity.

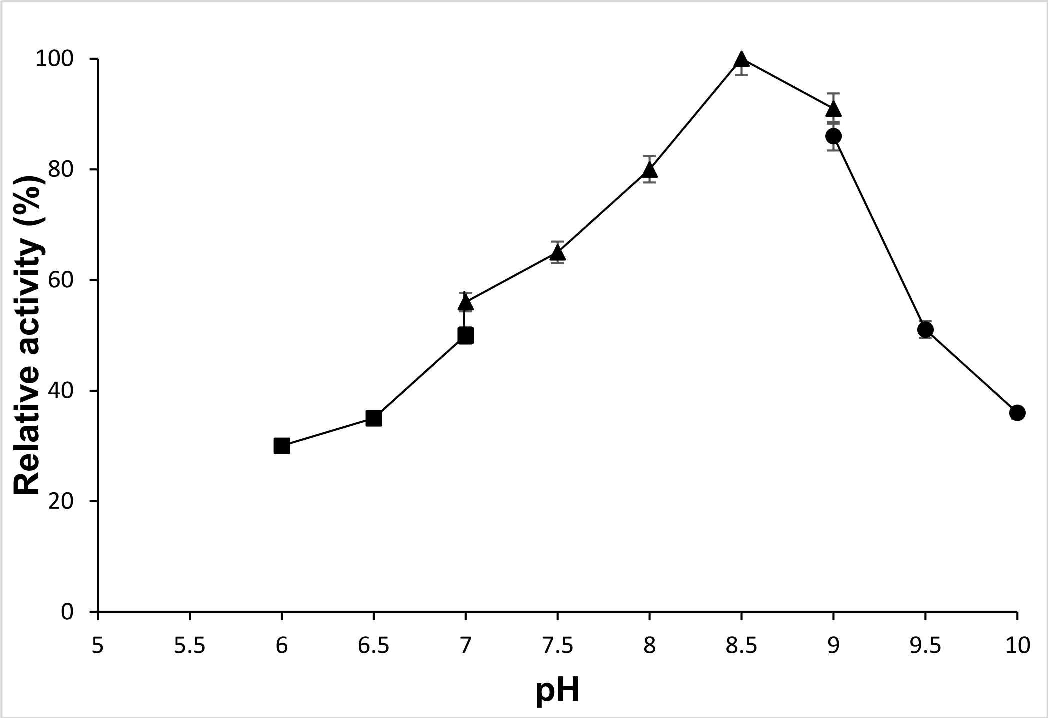

The activity assays were conducted at 55 °C in triplicates using Na-phosphate buffer (pH 6.0–7.0, rectangles); Tris-HCl buffer (pH 7.0–9.0, triangles) and Na-bicarbonate buffer (pH 9.0–10, circles).
